# Phenotypic plasticity in anti-intraguild predator strategies: mite larvae adjust their behaviours according to vulnerability and predation risk

**DOI:** 10.1007/s10493-012-9624-z

**Published:** 2012-10-28

**Authors:** Andreas Walzer, Peter Schausberger

**Affiliations:** Group of Arthropod Ecology and Behavior, Division of Plant Protection, Department of Crop Sciences, University of Natural Resources and Life Sciences, Peter Jordanstrasse 82, 1190 Vienna, Austria

**Keywords:** EthoVision, Inducible defences, Innate species recognition, Risk-sensitive behaviour, Intraguild interactions, Phytoseiidae

## Abstract

Interspecific threat-sensitivity allows prey to maximize the net benefit of antipredator strategies by adjusting the type and intensity of their response to the level of predation risk. This is well documented for classical prey-predator interactions but less so for intraguild predation (IGP). We examined threat-sensitivity in antipredator behaviour of larvae in a predatory mite guild sharing spider mites as prey. The guild consisted of the highly vulnerable intraguild (IG) prey and weak IG predator *Phytoseiulus persimilis*, the moderately vulnerable IG prey and moderate IG predator *Neoseiulus californicus* and the little vulnerable IG prey and strong IG predator *Amblyseius andersoni*. We videotaped the behaviour of the IG prey larvae of the three species in presence of either a low- or a high-risk IG predator female or predator absence and analysed time, distance, path shape and interaction parameters of predators and prey. The least vulnerable IG prey *A. andersoni* was insensitive to differing IGP risks but the moderately vulnerable IG prey *N. californicus* and the highly vulnerable IG prey *P. persimilis* responded in a threat-sensitive manner. Predator presence triggered threat-sensitive behavioural changes in one out of ten measured traits in *N. californicus* larvae but in four traits in *P. persimilis* larvae. Low-risk IG predator presence induced a typical escape response in *P. persimilis* larvae, whereas they reduced their activity in the high-risk IG predator presence. We argue that interspecific threat-sensitivity may promote co-existence of IG predators and IG prey and should be common in predator guilds with long co-evolutionary history.

## Introduction

Intraguild predation (IGP), the killing of food competitors, is a widespread phenomenon in both terrestrial and aquatic ecosystems (Polis et al. 1989; Arim and Marquet 2004; Sergio and Hiraldo 2008; Irigoien and de Roos 2011). IGP is well-studied from the perspective of the predator but poorly understood from the perspective of the prey. The occurrence and intensity of IGP commonly depend on the involved life stages (Polis et al. 1989) with individual guild members often reversing roles during ontogeny, from juvenile intraguild (IG) prey to adult IG predators (e.g., Magalhaes et al. 2005; Montserrat et al. 2011). Within guilds, IGP may be a major source of mortality for juvenile guild members (e.g. Walzer and Schausberger 1999a, b; Schausberger and Croft 2000a, b; Montserrat et al. 2011). Moreover, many IG prey species co-occur with multiple IG predator species posing different risks, ranging from insignificant to high IGP risk [mammalian carnivores (Schaller 1972; Hunter and Caro 2008; Glen et al. 2011); raptors (Lourenco et al. 2011); predatory insects (Rosenheim et al. 1993; Wissinger and McGrady 1993); mites (Walzer and Schausberger 2011a); tadpoles (Hawley 2009); salamander larvae (Gustafson 1993)]. Therefore, analogous to classical predation, to optimally balance the trade-offs between anti-IG predator behaviours and other fitness related activities such as foraging and mating, selection should favour IG prey, which is able to respond in an interspecific threat-sensitive manner to varying IGP risks (Walzer and Schausberger 2011a; Sih 1982; Helfman 1989).

Interspecific threat-sensitive antipredator responses are complex processes consisting of three phases: predator species recognition, assessment of the species-specific predation risk and the threat-sensitive antipredator response. All these phases depend on the life stages of IG predator and prey, and may be influenced by cue availability (single or multiple, direct, indirect, on body, left on substrate) and sensory modality (chemosensory, auditory, mechanosensory, visual, etc.), the diet of the IG predator and experience of IG predator and prey (e.g. Walzer and Schausberger 2011a). Predation risk of juvenile IG prey may be reduced by their mothers through oviposition site selection or killing of potential future IG predators (e.g. Walzer and Schausberger 2011a) and/or by themselves (analogous to cannibalism, e.g. Schausberger 2003). Usually it is a combination of both but in species without extended parental care juveniles should be threat-sensitive too because maternal influence is restricted to the time of egg laying yet the predation risk may change until egg hatch. While interspecific threat-sensitive anti-predator responses by classical prey are well documented for various taxa such as mammals (Blumstein et al. 2008; Monclus et al. 2009), birds (Edelaar and Wright 2006), fishes (Botham et al. 2008), lizards (Stapley 2003), amphibians (Kiesacker et al. 1996), and arthropods (Gyssels and Stoks 2005), evidence for interspecific threat-sensitive anti-IG predator responses is scarce and only available for maternal manipulation of offspring predation risk (Walzer and Schausberger 2011a). The peculiarities of IGP such as the low profitability of prey and the low predator–prey encounter rates compared to classical prey (Walzer and Schausberger 2011a) do not allow to simply extrapolate the concept of threat-sensitivity developed for classical predator–prey interactions (Sih 1982; Helfman 1989) to IGP. Studies on threat-sensitive anti-IG predator behaviours are important to understand the evolution of IGP and its consequences at the population and community levels, especially regarding the factors allowing IG predators to co-exist or not (Heithaus 2001; Amarasekare 2008; Urbani and Ramos-Jiliberto 2010).

Here, we studied interspecific IG predator recognition by juvenile IG prey within a natural guild of three plant-inhabiting predatory mite species, *Phytoseiulus persimilis*, *Neoseiulus californicus* and *Amblyseius andersoni* (Acari: Phytoseiidae). These predators co-occur in the Mediterranean region (personal observations, De Moraes et al. 2004) and interact with each other via competition for herbivorous mites, such as the two-spotted spider mite *Tetranychus urticae* (Acari: Tetranychidae), and mutual IGP (Schausberger and Croft 1999, 2000a, b; Walzer and Schausberger 2011a). They differ in adaptation to and strength in IGP and competition for spider mites. *Phytoseiulus persimilis* is a highly specialized predator of spider mites producing dense webbings and weak IG predator; *N. californicus* is a generalist predator with a preference for spider mites and an intermediate IG predator; *A. andersoni* is a broad generalist poorly adapted to utilize *T. urticae* as prey but a strong IG predator (McMurtry and Croft 1997; Schausberger and Croft 2000a; Walzer and Schausberger 1999a, b, 2011a, b). The small and little mobile larva is the most preferred IG prey of the larger IG predator females in phytoseid mites in general (Schausberger and Croft 2000a; Walzer and Schausberger 1999a, b, 2011a). We hypothesized that the larvae should be able to discriminate between different IG predator species and adjust their behaviours according to their vulnerability in IGP and the relative IGP risk posed by the other two species (little vulnerable *A. andersoni* with high risk *N. californicus* and low risk *P. persimilis;* moderately vulnerable *N. californicus* with high risk *A. andersoni* and low risk *P. persimilis;* highly vulnerable *P. persimilis* with high risk *A. andersoni* and low risk *N. californicus;* Walzer and Schausberger 2011a). To this end, we videotaped the behaviour of single larval IG prey on bean leaf discs in the presence or absence of a high- or low-risk IG predator female in each prey-predator combination and subsequently analysed predator and prey behaviours using EthoVision Pro^®^.

## Methods

### Species origin and rearing

*Phytoseiulus persimilis*, *N. californicus* and *A. andersoni* constitute a natural guild in Sicily and elsewhere in the Mediterranean basin (personal observations; De Moraes et al. 2004). Specimens of the three species used to found populations maintained in the laboratory were collected in the region Trapani, Sicily in 2007. In the laboratory, the species were separately reared on arenas consisting of plastic tiles resting on water-saturated foam cubes in plastic boxes half-filled with water and fed with *T. urticae* reared on common bean plants, *Phaseolus vulgaris* (see Walzer and Schausberger 2011a for details). To obtain similarly aged IG prey eggs giving rise to larvae used in experiments, gravid *A. andersoni*, *N. californicus* or *P. persimilis* females were randomly withdrawn from the rearing units and placed on detached bean leaves with surplus spider mite prey. After 2 h, the females were removed but their eggs were left on the arena. After 48 h all hatched larvae were removed and after further 4 h the newly hatched larvae, which were less than 4 h old, were collected and used as IG prey in the experiments.

### Experimental treatments, units and procedures

To imitate prey-predator interactions under natural conditions, larvae of each IG prey species were singly placed on leaf discs and exposed to either a low-risk IG predator or a high-risk IG predator or left without any predator, resulting in nine treatments. Leaf discs (diameter 14 mm) were punched out from the centre of detached trifoliate bean leaves including the mid vein. The chosen leaf disc size provided ample space for free movement of prey (body length ~0.2 mm) and predator (~0.5 mm), but at the same time boosted the likelihood of physical encounters. Each leaf disc was placed on the surface of a water column in an acrylic glass cylinder (height 20 mm, inner diameter 16 mm) leaving a ~1 mm wide water film barrier between the leaf edge and the inner margin of the acrylic glass cylinder, preventing the mites from escaping. After placing IG prey larvae on leaf discs they were allowed to acclimatize for ~10 min. To start the experiment, a single gravid IG predator female, randomly taken from the rearing unit and starved for 12 h before the experiment, was added onto the leaf disc—or the larva was left without a predator—and the behaviours of IG prey and predator were videotaped over 15 min. To identify behavioural changes over time, each behavioural parameter was separately computed for three consecutive time periods (0–5, 5–10, and 10–15 min).

### Video-taping and -tracking

The behaviour of the mites was videotaped with an analogue camera (Panasonic colour CCTV camera, model WV-CL 920A/G) with a 50 mm lens and a red photographic filter. The camera was fixed vertically to a binocular above the experimental units. The video signal was fed into a computerised video tracking system consisting of a personal computer equipped with a frame grabber (HaSoTec, FG-33-II) and the software EthoVision Pro^®^, version 3.1 (Noldus et al. 2001). To increase the contrast between the mites and the leaf surface (the background in the video), fluorescent, magenta coloured powder (Kurt Wolf & CO OHG, Vienna, Austria) was dusted on the dorsum of the mites and the videos shot under UV lighting. The powder did not affect the behaviour of the mites (personal observations). In the video the mites then appeared as large (IG predator) and small (IG prey) bright dots moving on a dark grey background. Grey scaling was used as detection method. The sampling rate was 3.5 samples per second, which was a compromise of the requirements on the highest possible sample rate, the processor speed and the storage capacity of the computer (Bell 1991). For analyses, each leaf disc arena was in-video subdivided into a leaf margin zone (a 1 mm wide ring along the edge of the leaf disc), a refuge zone (including the mid vein and a ~0.5 mm strip on the left and right from its longitudinal axis), and an open space zone (the area left and right from the mid vein without the refuge and margin zone). The leaf margin is a zone of high predator activity because of the edge-oriented searching behaviour of many phytoseiid predators (e.g. Sabelis and Dicke 1985). The vicinity of leaf veins is used by many phytoseiid larvae as a refuge from biotic and abiotic hazards (e.g. Norton et al. 2001).

### Time, distance and path shape parameters

Four time and distance parameters (time spent in each zone, total distance moved, mean velocity and activity) and three path shape parameters (absolute turning angle, absolute angular velocity and absolute meander) were computed from track data using EthoVision Pro^®^ (for detailed algorithms of parameter calculation see Noldus Information Technology, 2005). Time spent in zones is the time spent by the mites in the margin, refuge and open space zones in seconds (s). Total distance moved is the total length of the path moved by the mites (mm). Mean velocity is the average speed at which the mites moved (mm/s). Activity is the time (s) spent moving. Absolute turning angle (°) is the absolute change in direction of a moving mite and corresponds to the difference in direction of an individual’s movement between two consecutive samples. Absolute angular velocity (°/s) is calculated by dividing the turning angle by the sample interval and is an indicator of how fast an object is changing its direction. Absolute meander (°/mm) is the turning angle divided by the distance moved, which gives an estimation of the level of path tortuosity (Bell 1991). All three path shape parameters range from 0° to 180°. To prevent small movements caused by noise of the system or so-called pivoting on the spot to be scored as genuine movement by the mites, a minimum distance filter of 0.5 mm was used in the analyses of all time and distance parameters except for time spent in zones. By that way, scoring took only place when the object had moved 0.5 mm away from the point at which the parameter had been scored the previous time.

### Interactions between IG prey and IG predators

The parameters proximity and relative movement were calculated without applying the minimum distance filter. Proximity is defined as the state in which the IG prey and the IG predator are within such a distance from each other that the IG predator is able to touch the IG prey when facing each other. Proximity is scored when the “center of gravity” (the middle of the dorsal shield) of IG predator and IG prey are within a predefined distance. The predefined distance was calculated for each IG prey/predator pairing by summing up half the dorsal shield length and the length of the first pair of legs of both IG prey and IG predator. The morphometric data were used from Croft et al. (1999). Relative movement is the relative displacement between IG prey and predator, whereby the speed and direction of the movement of both IG prey and predator are taken into account. This allows distinguishing between relative movements of an object (IG prey or predator) to or from the other object. Movement by the IG prey away from the IG predator may be interpreted as avoidance response. Movements by the IG predator towards the IG prey may be interpreted as approaching behaviour.

### Statistical analyses

Separate statistical analyses were carried out for each IG prey species using SPSS 15.0.1 (SPSS Inc., Chicago, USA, 2006). Generalised estimating equations (GEE, normal distribution with identity link function, autocorrelation structure between observation periods) were used to compare the influence of IG predator species (no, low, or high risk for IG prey; low or high risk for IG predator) on time, distance, path shape and interaction parameters of IG prey and predators over time (three observation periods used as within subject variable). The residence time of IG prey and IG predators were analysed only for the zones leaf margin and refuge. All proportional parameters (residence time, activity, spatial proximity, IG predator avoidance, IG predator approaching) were arcsin square root transformed before analyses. To detail changes over time within each IG prey and IG predator species and differences among predator species within IG prey species, the estimated marginal means were compared by least significant difference (LSD) tests if needed. All individuals, the survivors and those that died during the experiment, were included in GEEs. The behaviours of individuals that died during the experiment were only scored and computed for those 5 min periods where they stayed alive throughout this period. Before performing GEEs, we analysed the effects of predator species (no, low or high risk) on IG prey behaviour performed during the initial 5 min by generalised linear models (GLM, normal distribution, identity link) including IG prey state (survived or died between 5 min and the end of the experiment) as covariate. The only purpose of these GLMs was to exclude that differences in IG prey behaviours among predator species (no, low risk, high risk) were due to selective predation and escaping and thus merely reflect innate individual differences but not true antipredator responses—assuming that differences among IG prey exposed to no, low or high risk could be due to the fact that individuals with a given innate behaviour survive more likely than others.

## Results

Preliminary GLMs revealed that in neither IG prey species and neither parameter prey state (survived vs. killed/escaped during the 15 min experiment) had a significant effect (*P* > 0.05) on IG prey behaviour indicating that killed and escaped IG prey individuals responded in a similar manner to predator presence as surviving IG prey individuals. Thus, the covariate prey state was excluded from GEEs. All subsequently reported statistical results were generated by GEEs for the total experimental period including both survived and killed/escaped IG prey individuals.

### IG prey *Amblyseius andersoni*

#### Time, distance and path shape parameters

**Fig. 1:**
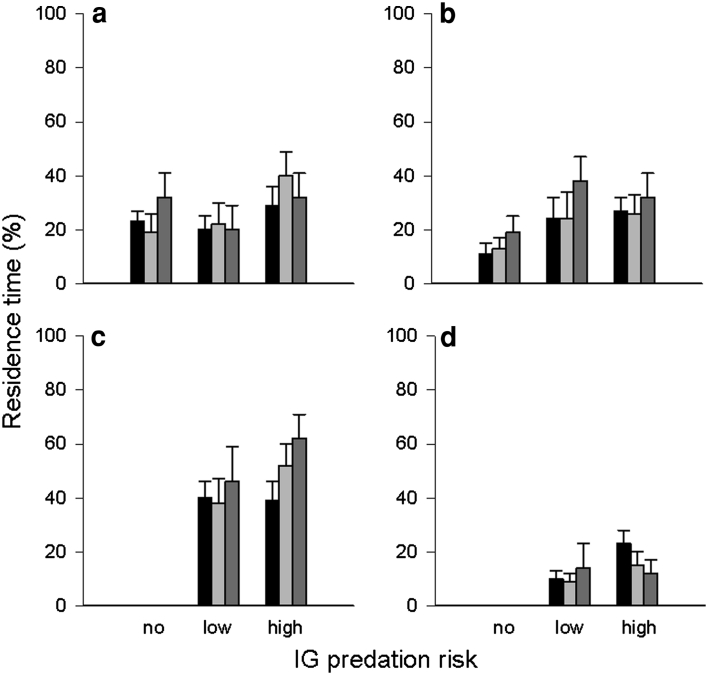
Influence of IG predator species (no, low, or high risk for IG prey; low or high risk for IG predator) on percent residence time (mean + SE) of the IG prey *Amlyseius andersoni* (**a**, **b**) and the IG predators *Phytoseiulus persimilis* (low risk) and *Neoseiulus californicus* (high risk) (**c**, **d**) in the leaf margin (**a**, **c**) and refuge (**b**, **d**) over time (0–5 min: *black bars*, 5–10 min: *light grey bars*, 10–15 min: *dark grey bars*)

The residence time of IG prey and IG predators in the leaf margin and refuge was neither affected by IG predator species (IG prey: leaf margin: *Wald x*_2_^2^ = 2.109, *P* = 0.348; refuge: *Wald x*_2_^2^ = 4.482, *P* = 0.106; IG predator: leaf margin: *Wald x*_1_^2^ = 0.932, *P* = 0.334; refuge: *Wald x*_1_^2^ = 0.740, *P* = 0.390), time (IG prey: leaf margin: *Wald x*_2_^2^ = 0.246, *P* = 0.884; refuge: *Wald x*_2_^2^ = 2.942, *P* = 0.230; IG predator: leaf margin: *Wald x*_2_^2^ = 4.392, *P* = 0.111; refuge: *Wald x*_2_^2^ = 4.018, *P* = 0.134), nor their interaction (IG prey: leaf margin: *Wald x*_4_^2^ = 7.790, *P* = 0.100; refuge: *Wald x*_4_^2^ = 1.434, *P* = 0.838; IG predator: leaf margin: *Wald x*_2_^2^ = 2.416, *P* = 0.229; refuge: *Wald x*_2_^2^ = 2.104, *P* = 0.349) (Fig. 1a–d).

**Fig. 2:**
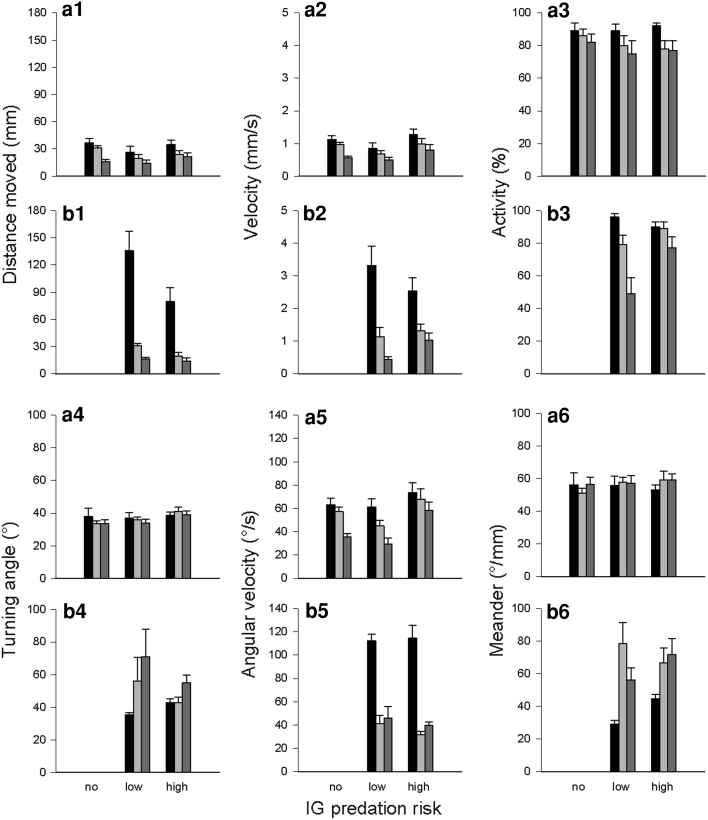
Influence of IG predator species [no, low, or high risk for IG prey (**a**); low or high risk for IG predator (**b**)] on distance moved (1), velocity (2), activity (3), absolute turning angle (4), absolute angular velocity (5) and absolute meander (6) (mean + SE) of the IG prey *Amblyseius andersoni* and the IG predators *Phytoseiulus persimilis* (low risk) and *Neoseiulus californicus* (high risk) over time (0–5 min: *black bars*, 5–10 min: *light grey bars*, 10–15 min: *dark grey bars*)

The distance moved decreased over time in both IG predators and IG prey. However, within the 1st time period (0–5 min) the low-risk IG predator covered a longer distance than the high-risk IG predator (*P* = 0.032). Conversely, IG prey covered a shorter distance in the 2nd time period in presence of the low-risk predator than in predator absence (no vs. low risk: *P* = 0.019; no vs. high risk: *P* = 0.171; low vs. high risk: *P* = 0.469) (Table 1; Fig. 2a1, b1). Irrespective of predator species, both IG predators and IG prey reduced the running velocity over time (*P* < 0.001 for every pairwise comparison) (Table 1; Fig. 2a2, b2), and their activity decreased over time (IG prey: 1st vs. 3rd time period: *P* = 0.004; IG predators: 1st vs. 3rd: *P* < 0.001). Within the 3rd time period (10–15 min) the high-risk predator was more active than the low-risk predator (*P* = 0.027) (Table 1; Fig. 2a3, b3).

**Table 1:** Generalised estimating equations (GEE, normal distribution with identity link function, autocorrelation structure between observation periods) for the influence of IG predator species (no, low, or high risk for IG prey; low or high risk for IG predator) on time, distance and path shape parameters of the IG prey *Amblyseius andersoni* and IG predators *Phytoseiulus persimilis* (low risk) and *Neoseiulus californicus* (high risk) over time (three observation periods: 0–5, 5–10, 10–15 min)

Parameter	Factor	IG prey			IG predator		
		Wald x^2^	*df*	*P*	Wald x^2^	*df*	*P*
DM (mm)	Time	36.492	2	<0.001	56.738	2	<0.001
	IG predator species	2.344	2	0.310	1.471	1	0.225
	Interaction	13.371	4	0.010	8.947	2	0.011
V (mm/s)	Time	52.060	2	<0.001	46.440	2	<0.001
	IG predator species	3.966	2	0.138	0.001	1	0.991
	Interaction	8.102	4	0.088	4.270	2	0.118
A (%)	Time	11.279	2	0.004	24.824	2	<0.001
	IG predator species	1.309	2	0.502	4.244	1	0.039
	Interaction	2.613	4	0.624	10.963	2	0.004
ATA (°)	Time	1.841	2	0.398	4.608	2	0.100
	IG predator species	3.287	2	0.193	0.251	1	0.616
	Interaction	4.574	4	0.334	3.641	2	0.162
AAV (°/s)	Time	53.855	2	<0.001	46.141	2	<0.001
	IG predator species	6.147	2	0.046	0.041	1	0.839
	Interaction	5.116	4	0.276	2.898	2	0.235
AM (mm/s)	Time	0.596	2	0.742	12.052	2	0.002
	IG predator species	0.265	2	0.876	0.174	1	0.677
	Interaction	5.739	4	0.219	3.610	2	0.165

*DM* distance moved, *V* velocity, *A* activity, *ATA* absolute turning angle, *AAV* absolute angular velocity, *AM* absolute meander

The absolute turning angles of IG prey and IG predators were neither affected by the IG predator species, time, nor their interaction (Table 1; Fig. 2a4, b4). The absolute angular velocity of both IG prey and IG predators and the absolute meander of the IG predators differed among predator species (Table 1). Angular velocity of IG prey decreased over time but the presence of the high-risk IG predator triggered faster changes of direction than the presence of the low-risk IG predator (no vs. low risk: *P* = 0.230; low vs. high risk: *P* = 0.013; no vs. high risk: *P* = 0.071). Irrespective of IG predator species, angular velocity of the IG predators was lower in the 2nd and 3rd time period (1st vs. 2nd: *P* < 0.001; 1st vs. 3rd: *P* < 0.001; 2nd vs. 3rd: *P* = 0.276) (Table 1; Fig. 2a5, b5), but the IG predators meandered significantly more in the 3rd period (1st vs. 2nd: *P* = 0.104; 1st vs. 3rd: *P* = 0.003; 2nd vs. 3rd: *P* = 0.002) (Table 1; Fig. 2a6, b6).

#### Interactions between IG prey and IG predators

**Fig. 3:**
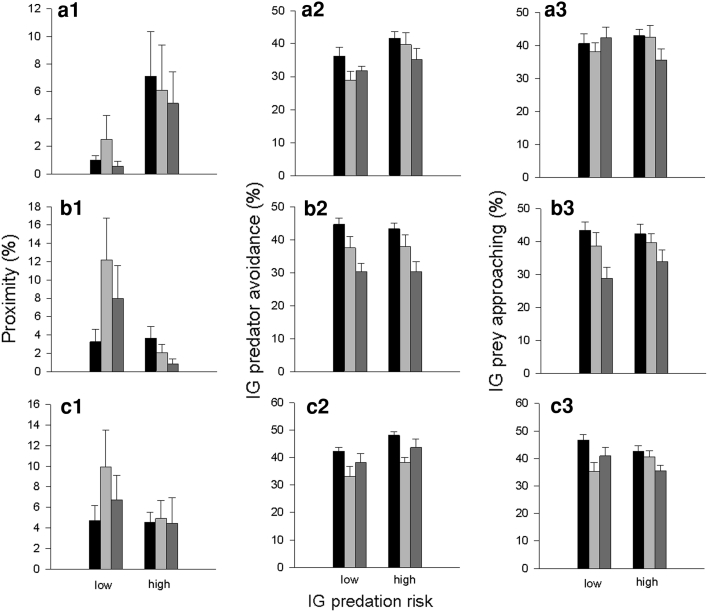
Influence of IG predator species (low or high risk for IG prey and IG predator) on the spatial proximity between IG prey and IG predators (1), IG predator avoidance by IG prey (2), and IG prey approaching by IG predator (3) (mean + SE) over time (0–5 min: *black bars*, 5–10 min: *light grey bars*, 10–15 min: *dark grey bars*). **a** IG prey *Amblyseius andersoni,* low-risk predator *Phytoseiulus persimilis*, high-risk predator *Neoseiulus californicus*; **b** IG prey *N. californicus*, low-risk predator *P. persimilis,* high-risk predator *A. andersoni*; **c** IG prey *P. persimilis,* low-risk predator *N. californicus*, high-risk predator *A. andersoni*

Intraguild prey spent marginally more time in the proximity of the high- than low-risk IG predator (GEE; *Wald x*_1_^2^ = 3.369, *P* = 0.066). Time (*Wald x*_2_^2^ = 3.092, *P* = 0.213) and the interaction of predator species and time (*Wald x*_2_^2^ = 1.791, *P* = 0.408) did not influence the duration of proximity between IG prey and IG predators (Fig. 3a1). IG prey spent more time moving away from the high-risk IG predator (IG predator species: *Wald x*_1_^2^ = 4.795, *P* = 0.029; IG predator species*time: *Wald x*_2_^2^ = 3.291, *P* = 0.193). The avoidance response decreased marginally over time (*Wald x*_2_^2^ = 5.368, *P* = 0.068; 1st vs. 2nd: *P* = 0.043; 1st vs. 3rd: *P* = 0.034; 2nd vs. 3rd: *P* = 0.763) (Fig. 3b1). Time (*Wald x*_2_^2^ = 0.999, *P* = 0.607) and IG predator species (*Wald x*_1_^2^ = 0.010, *P* = 0.921) did not influence the time spent by IG predators approaching IG prey. However, the interaction between the main factors was marginally significant (*Wald x*_2_^2^ = 5.301, *P* = 0.071). The approaching behaviour of the low-risk IG predator did not change over time (1st vs. 2nd period: *P* = 0.531; 1st vs. 3rd: *P* = 0.715; 2nd vs. 3rd: *P* = 0.523) but decreased over time in the high-risk IG predator (1st vs. 3rd: *P* = 0.017) (Fig. 3c1).

### IG prey *Neoseiulus californicus*

#### Time, distance and path shape parameters

**Fig. 4:**
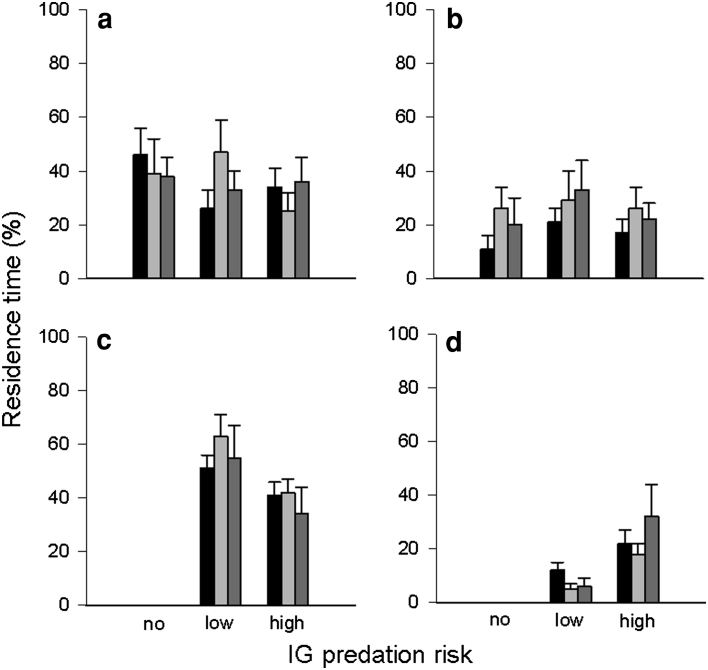
Influence of IG predator species (no, low, or high risk for IG prey; low or high risk for IG predator) on percent residence time (mean + SE) of the IG prey *Neoseiulus californicus* (**a**, **b**) and the IG predators *Phytoseiulus persimilis* (low risk) and *Amblyseius andersoni* (high risk) (**c**, **d**) in the leaf margin (**a**, **c**) and refuge (**b**, **d**) over time (0–5 min: *black bars*, 5–10 min: *light grey bars*, 10–15 min: *dark grey bars*)

The residence time of IG prey in the leaf margin and refuge was not influenced by IG predator species (leaf margin: *Wald x*_2_^2^ = 0.588, *P* = 0.745; refuge: *Wald x*_2_^2^ = 0.747, *P* = 0.688) and time (leaf margin: *Wald x*_2_^2^ = 0.060, *P* = 0.970; refuge: *Wald x*_2_^2^ = 2.320, *P* = 0.313) (Fig. 4a, b). The interaction between IG predator species and time was marginally significant for the residence time of IG prey in the leaf margin (*Wald x*_4_^2^ = 9.132, *P* = 0.058), but not for the refuge (*Wald x*_4_^2^ = 3.627, *P* = 0.459). IG prey spent more time in the leaf margin in the 2nd time period (5–10 min) than in the other periods in the presence of the low-risk IG predator (0–5 min vs. 5–10 min: *P* = 0.091; 0–5 min vs. 10–15 min: *P* = 0.642; 5–10 min vs. 10–15 min: *P* = 0.023) (Fig. 4a, b). Irrespective of time, the low-risk IG predator spent more time in the leaf margin than the high-risk predator (time: *Wald x*_2_^2^ = 4.258, *P* = 0.119; IG predator species: *Wald x*_1_^2^ = 5.506, *P* = 0.019; IG predator species*time: *Wald x*_2_^2^ = 1.527, *P* = 0.466). The residence time of the IG predators in the refuge was affected by both IG predator species (*Wald x*_1_^2^ = 12.461, *P* < 0.001) and time (*Wald x*_2_^2^ = 26.996, *P* < 0.001) but not by their interaction (*Wald x*_2_^2^ = 2.136, *P* = 0.344). The high-risk IG predator spent more time in the refuge than the low-risk IG predator. Both IG predators remained longer in the refuge in the 1st than 2nd time period (1st vs. 2nd: *P* < 0.001; 1st vs. 3rd: *P* = 0.713; 2nd vs. 3rd: *P* = 0.425) (Fig. 4c, d).

**Fig. 5:**
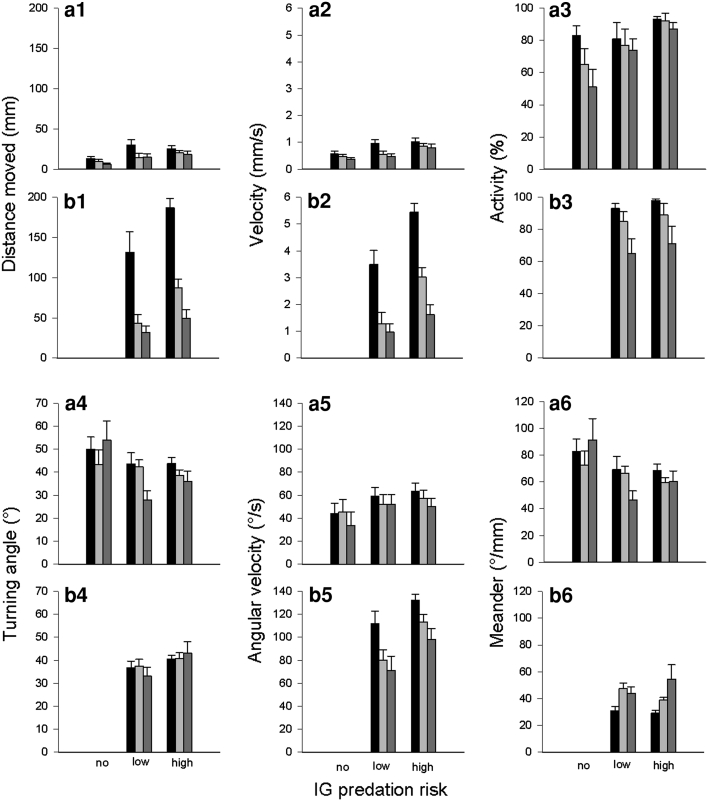
Influence of IG predator species [no, low, or high risk for IG prey (**a**); low or high risk for IG predator (**b**)] on distance moved (1), velocity (2), activity (3), absolute turning angle (4), absolute angular velocity (5) and absolute meander (6) (mean + SE) of the IG prey *Neoseiulus californicus* and IG predators *Phytoseiulus persimilis* (low risk) and *Amblyseius andersoni* (high risk) over time (0–5 min: *black bars*, 5–10 min: *light grey bars*, 10–15 min: *dark grey bars*)

Irrespective of predator species, the distance moved and velocity of IG prey decreased over time (distance: 1st vs. 3rd time period: *P* = 0.001; velocity: 1st vs. 3rd: *P* < 0.001). The presence of the high-risk IG predator triggered an increase in both the covered distance and velocity of IG prey, whereas the presence of the low-risk predator resulted only in a longer distance moved by IG prey (distance: no vs. low risk: *P* = 0.020; no vs. high risk: *P* = 0.001; low vs. high risk: *P* = 0.770; velocity: no vs. low risk: *P* = 0.144; no vs. high risk: *P* = 0.003; low vs. high risk: *P* = 0.132) (Table 2; Fig. 5a1, a2). The distance moved and velocity of both IG predators decreased over time but both parameters were higher in the high-risk IG predator (Table 2; Fig. 5b1, b2). Activity of IG prey and IG predators decreased over time (IG prey: 1st vs. 3rd time period: *P* = 0.002; IG predators: 1st vs. 3rd: *P* < 0.001). IG prey was more active in the presence than absence of the high-risk IG predator (no vs. low risk: *P* = 0.339; no vs. high risk: *P* = 0.003; low vs. high risk: *P* = 0.121). Pooled over time, the high-risk IG predator was marginally significantly more active than the low-risk IG predator (Table 2; Fig. 5a3, b3).

**Table 2:** Generalised estimating equations (GEE, normal distribution, identity link function, autocorrelation structure between observation periods) for the influence of IG predator species (no, low, or high risk for IG prey; low or high risk for IG predator) on time, distance and path shape parameters of the IG prey *Neoseiulus californicus* and IG predators *Phytoseiulus persimilis* (low risk) and *Amblyseius andersoni* (high risk) over time (three observation periods: 0–5, 5–10, 10–15 min)

Parameter	Factor	IG prey			IG predator		
		Wald x^2^	*df*	*P*	Wald x^2^	*df*	*P*
DM (mm)	Time	13.175	2	0.001	59.768	2	<0.001
	IG predator species	13.535	2	0.001	8.80	1	0.003
	Interaction	3.779	4	0.437	5.758	2	0.056
V (mm/s)	Time	21.605	2	<0.001	69.230	2	<0.001
	IG predator species	9.261	2	0.010	9.626	1	0.002
	Interaction	4.169	4	0.384	13.074	2	0.001
A (%)	Time	10.266	2	0.006	16.193	2	<0.001
	IG predator species	9.951	2	0.007	2.738	1	0.098
	Interaction	5.302	4	0.258	0.151	2	0.927
ATA (°)	Time	3.656	2	0.161	0.070	2	0.966
	IG predator species	7.113	2	0.029	4.016	1	0.045
	Interaction	7.104	4	0.131	0.735	2	0.692
AAV (°/s)	Time	16.850	2	<0.001	20.535	2	0.006
	IG predator species	3.382	2	0.184	7.561	1	0.006
	Interaction	8.025	4	0.091	1.313	2	0.519
AM (mm/s)	Time	3.527	2	0.171	33.832	2	<0.001
	IG predator species	6.800	2	0.033	0.001	1	0.996
	Interaction	5.272	4	0.171	2.662	2	0.284

*DM* distance moved, *V* velocity, *A* activity, *ATA* absolute turning angle, *AAV* absolute angular velocity, *AM* absolute meander

Irrespective of time, turning angle and meander of IG prey were lower in presence than absence of the IG predators (turning angle: no vs. low risk: *P* = 0.008, no vs. high risk: *P* = 0.014, low vs. high risk: *P* = 0.602; meander: no vs. low risk: *P* = 0.015, no vs. high risk: *P* = 0.014, low vs. high risk: *P* = 0.643). Irrespective of predator species, the angular velocity of IG prey decreased in the 3rd time period (1st vs. 2nd: *P* = 0.374; 1st vs. 3rd and 2nd vs. 3rd: *P* < 0.001) (Table 2; Fig. 5a4, a5, a6). The absolute turning angle of the IG predators did not change over time but was higher in the high-risk IG predator across time (Fig. 5b4). Absolute angular velocity of both IG predators decreased over time (1st vs. 3rd time period: *P* < 0.001) and was higher in the high-risk IG predator across time (Fig. 5b5). Both IG predators meandered more in the 2nd and 3rd time period (1st vs. 2nd: *P* < 0.001; 1st vs. 3rd: *P* = 0.002; 2nd vs. 3rd: *P* = 0.361) (Table 2; Fig. 5b6).

#### Interactions between IG prey and IG predators

Intraguild prey spent less time in the proximity of the high than low-risk IG predator (*Wald x*_1_^2^ = 14.214, *P* < 0.001). However, this effect depended on time (time: *Wald x*_2_^2^ = 1.828, *P* = 0.401; IG predator species*time: *Wald x*_2_^2^ = 8.926, *P* = 0.016). The duration of time spent by IG prey in proximity of the low-risk IG predator increased over time (1st vs. 2nd: *P* = 0.041). The opposite trend was observed for proximity between IG prey and the high risk IG predator (1st vs. 3rd time period: *P* = 0.005) (Fig. 3b1). Both the time spent by IG prey in moving away from the IG predators and the time spent by the IG predators in approaching the IG prey decreased over time (IG prey: time: *Wald x*_2_^2^ = 38.723, *P* < 0.001; IG predator species: *Wald x*_2_^2^ = 0.038, *P* = 0.845; IG predator species*time: *Wald x*_2_^2^ = 0.076, *P* = 0.963; IG predators: time: *Wald x*_2_^2^ = 21.151, *P* < 0.001; IG predator species: *Wald x*_1_^2^ = 0.271, *P* = 0.603; IG predator species*time: *Wald x*_2_^2^ = 1.650, *P* = 0.438) (Fig. 3b2, b3).

### IG prey *Phytoseiulus persimilis*

#### Time, distance and path shape parameters

**Fig. 6:**
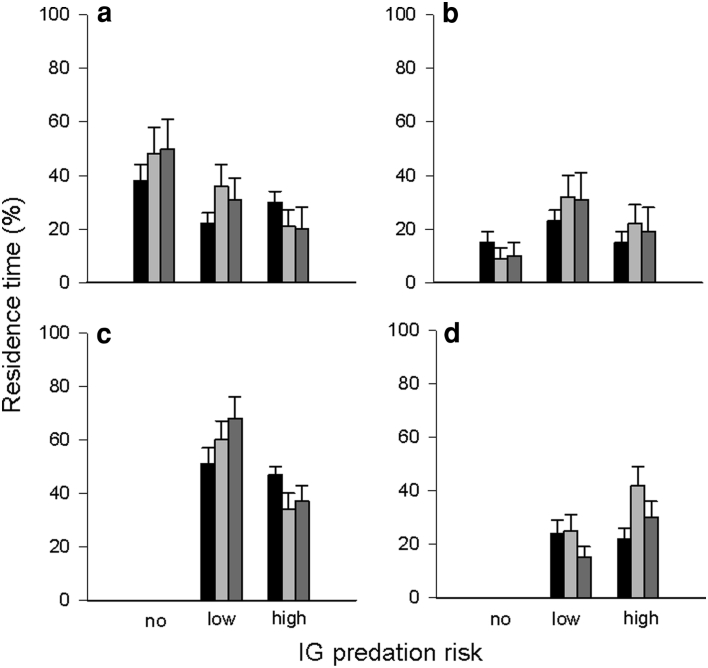
Influence of IG predator species (no, low, or high risk for IG prey; low or high risk for IG predator) on percent residence time (mean + SE) of the IG prey *Phytoseiulus persimilis* (**a**, **b**) and the IG predators *Neoseiulus californicus* (low risk) and *Amblyseius andersoni* (high risk) (**c**, **d**) in the leaf margin (**a**, **c**) and refuge (B, D) over time (0–5 min: *black bars*, 5–10 min: *light grey bars*, 10–15 min: *dark grey bars*)

The residence duration of IG prey in the leaf margin and refuge was influenced by IG predator species (leaf margin: *Wald x*_2_^2^ = 6.373, *P* = 0.041; refuge: *Wald x*_2_^2^ = 9.032, *P* = 0.011) but not by time (leaf margin: *Wald x*_2_^2^ = 0.352, *P* = 0.839; refuge: *Wald x*_2_^2^ = 0.493, *P* = 0.782) and the interaction of time and IG predator species (leaf margin: *Wald x*_4_^2^ = 6.692, *P* = 0.153; refuge: *Wald x*_4_^2^ = 3.021, *P* = 0.554). IG prey spent more time in the leaf margin in absence than presence of the high-risk IG predator (no vs. low risk: *P* = 0.123; no vs. high risk: *P* = 0.013; low vs. high risk: *P* = 0.187). IG prey remained longer in the refuge in presence of the low-risk IG predator than predator absence (no vs. low risk: *P* = 0.003; no vs. high risk: *P* = 0.284; low vs. high risk: *P* = 0.126) (Fig. 6 a, b). The effect of IG predator species on their residence time in the leaf margin depended on time (IG predator species: *Wald x*_1_^2^ = 8.352, *P* = 0.004; time: *Wald x*_2_^2^ = 1.474, *P* = 0.479; IG predator species*time: *Wald x*_2_^2^ = 18.997, *P* < 0.001). The residence time of both IG predators in the leaf margin was similar in the 1st time period (*P* = 0.579). Afterwards, the low-risk IG predator spent more time in the leaf margin than the high-risk IG predator (2nd time period: *P* = 0.002; 3rd time period: *P* = 0.002). The residence time of the IG predators in the refuge was influenced by IG predator species (*Wald x*_1_^2^ = 3.566, *P* = 0.050), time (*Wald x*_2_^2^ = 13.346, *P* = 0.001) and their interaction (*Wald x*_2_^2^ = 10.269, *P* = 0.006). The residence time of the low-risk IG predator in the refuge decreased over time (1st vs. 3rd: *P* < 0.001). The residence time of the high-risk IG predator was longer in the 2nd time period than in the other periods (1st vs. 2nd: *P* < 0.001; 1st vs. 3rd: *P* = 0.439; 2nd vs. 3rd: *P* = 0.004) (Fig. 6c, d).

**Fig. 7:**
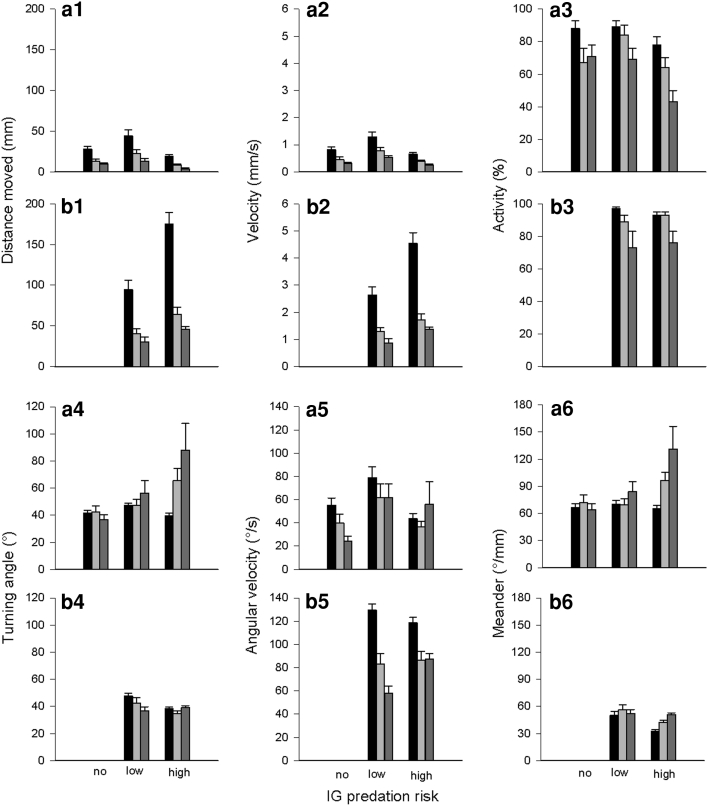
Influence of IG predator species [no, low, or high risk for IG prey (**a**); low or high risk for IG predator (**b**)] on distance moved (1), velocity (2), activity (3), absolute turning angle (4), absolute angular velocity (5) and absolute meander (6) (mean + SE) of the IG prey *Phytoseiulus persimilis* and the IG predators *Neoseiulus californicus* (low risk) and *Amblyseius andersoni* (high risk) over time (0–5 min: *black bars*, 5–10 min: *light grey bars*, 10–15 min: *dark grey bars*)

The distance moved and velocity of IG prey decreased over time (1st vs. 3rd time period: *P* < 0.001 for both parameters). In absence of the IG predators, IG prey covered a longer distance than in presence of the high-risk IG predator but a shorter distance than in presence of the low-risk IG predator (no vs. low risk: *P* = 0.034; no vs. high risk: *P* = 0.028; high vs. low risk: *P* < 0.001) (Table 3; Fig. 7a1). Velocity of IG prey did not differ between predator absence and presence of the high-risk IG predator (*P* = 0.250) but was higher in presence of the low-risk IG predator (no vs. low risk: *P* = 0.006; high vs. low risk: *P* < 0.001) (Table 3; Fig. 7a2). The distance moved and velocity decreased over time in both IG predator species. Both parameters were in total higher in the high-risk IG predator (Table 3; Fig. 7b1, b2). Irrespective of predator species, activity of IG prey decreased over time (1st vs. 3rd period: *P* < 0.001) and was the lowest in presence of the high-risk IG predator (no vs. low risk: *P* = 0.347; no vs. high risk: *P* = 0.014; low vs. high risk: *P* = 0.001). Irrespective of predator species, IG predator activity decreased continuously over time (1st vs. 3rd period: *P* < 0.001) (Table 3; Fig. 7a3, b3).

**Table 3:** Generalised estimating equations (GEE, normal distribution, identity link function, autocorrelation structure between observation periods) for the influence of IG predator species (no, low, or high risk for IG prey; low or high risk for IG predator) on time, distance and path shape parameters of the IG prey *Phytoseiulus persimilis* and IG predators *Neoseiulus californicus* (low risk) and *Amblyseius andersoni* (high risk) over time (three observation periods: 0–5, 5–10, 10–15 min)

Parameter	Factor	IG prey			IG predator		
		Wald x^2^	*df*	*P*	Wald x^2^	*df*	*P*
DM (mm)	Time	61.197	2	<0.001	134.307	2	<0.001
	IG predator species	19.046	2	<0.001	16.393	1	<0.001
	Interaction	6.127	4	0.190	15.116	2	0.001
V (mm/s)	Time	70.741	2	<0.001	144.082	2	<0.001
	IG predator species	17.765	2	<0.001	14.220	1	<0.001
	Interaction	4.829	4	0.305	12.893	2	0.002
A (%)	Time	29.991	2	<0.001	23.787	2	<0.001
	IG predator species	12.648	2	0.002	0.271	1	0.602
	Interaction	4.278	4	0.370	0.416	2	0.812
ATA (°)	Time	7.685	2	0.021	12.650	2	0.002
	IG predator species	11.000	2	0.004	3.891	1	0.049
	Interaction	11.609	4	0.021	12.941	2	0.002
AAV (°/s)	Time	13.008	2	0.001	135.682	2	<0.001
	IG predator species	7.940	2	0.019	1.245	1	0.265
	Interaction	5.831	4	0.212	18.878	2	<0.001
AM (mm/s)	Time	10.248	2	0.006	11.134	2	0.004
	IG predator species	8.309	2	0.016	9.902	1	0.002
	Interaction	12.960	4	0.011	6.928	2	0.031

*DM* distance moved, *V* velocity, *A* activity, *ATA* absolute turning angle, *AAV* absolute angular velocity, *AM* absolute meander

The absolute turning angle and meander of IG prey was constant over time in presence of the low-risk IG predator and predator absence (*P* > 0.05). IG prey meandered more and turned more strongly in the presence of the high-risk IG predator over time (turning angles: 1st vs. 3rd: *P* = 0.012; meander: 1st vs. 3rd: *P* = 0.007) (Table 3; Fig. 7a4, a6). Irrespective of predator species, the absolute angular velocity of IG prey decreased over time (1st vs. 3rd: *P* = 0.036). Pooled over time, angular velocity of IG prey was the highest in presence of the low-risk predator (no vs. low risk: *P* = 0.005, no vs. high risk: *P* = 0.550, low vs. high risk: *P* = 0.080) (Table 3; Fig. 7a5).The absolute turning angle of the high-risk IG predator did not change over time but was lower in the 1st time period than the turning angle of the low-risk IG predator (*P* < 0.001). Conversely, the turning angle of the low-risk IG predator decreased over time (1st vs. 3rd: *P* < 0.001) (Table 3; Fig. 7b4). The absolute angular velocity of both IG predators decreased over time (1st vs. 3rd time period: *P* < 0.001). In the 3rd time period angular velocity was higher in the high- than in the low-risk IG predator (*P* < 0.001) (Table 3; Fig. 7b5). The absolute meander did not change over time in the low-risk IG predator but increased over time in the high-risk IG predator (1st vs. 3rd time period: *P* < 0.001) (Table 3; Fig. 7b6).

#### Interactions between IG prey and IG predators

The time spent by IG prey in proximity of the IG predators was neither influenced by predator species (*Wald x*_1_^2^ = 0.679, *P* = 0.410), time (*Wald x*_2_^2^ = 0.656, *P* = 0.720), nor their interaction (*Wald x*_2_^2^ = 3.810, *P* = 0.149) (Fig. 3c1). However, avoidance behaviour of IG prey was more intense in the presence of the high risk IG predator (*Wald x*_1_^2^ = 5.392, *P* = 0.020). The time spent moving away from the IG predators by IG prey decreased over time (*Wald x*_2_^2^ = 23.194, *P* < 0.001; IG predator species*time: *Wald x*_2_^2^ = 0.006, *P* = 0.997) (Fig. 3c2). The approaching behaviour of the two IG predators was only affected by time (*Wald x*_2_^2^ = 10.755, *P* = 0.005), i.e. the time spent moving to the IG prey decreased over time (*P* < 0.05 for every pair wise comparison), but not predator species (*Wald x*_1_^2^ = 0.388, *P* = 0.561) and the interaction between time and predator species (*Wald x*_2_^2^ = 5.370, *P* = 0.068) (Fig. 3c3).

## Discussion

Our study suggests innate interspecific threat-sensitive anti-IG predator responses in two out of three acarine prey species. The level of investment in anti-IG predator behaviour by the three prey species corresponds to their degree of vulnerability to IGP, as reflected by the number of behavioural traits changed in response to predator presence. Only two out of ten behavioural traits of the least vulnerable IG prey *A. andersoni,* angular velocity and temporal IG predator avoidance, were influenced by IG predator presence but not by the threat posed by the predators. The moderately vulnerable IG prey *N. californicus* responded in six out of ten measured traits to predator presence. Threat-sensitivity was unambiguously apparent in the amount of time spent in predator proximity. The highly vulnerable IG prey *P. persimilis* responded in nine out of ten measured traits to predator presence but adopted different threat-sensitive strategies in dependence of predation risk. *P. persimilis* larvae were less active, moved shorter distances, decreased their running speed, turned faster and meandered more in presence of the high- than the low-risk IG predator and spent more time in avoiding the high-risk predator.

### Threat-sensitivity

A highly important aspect in assessments of inducible antipredator behaviours and threat-sensitivity is whether the predator is physically present and thus has the possibility to directly interact with prey or not (Lima 2002). The majority of studies dealing with interspecific threat-sensitive responses of classical prey used only predator cues (Kiesacker et al. 1996; Stapley 2003; Edelaar and Wright 2006; Blumstein et al. 2008) or caged predators (Botham et al. 2008) as predator stimuli (but see Monclus et al. 2009). Physical absence of the predator eases interpretation of threat-sensitivity but may give an incomplete picture of possible antipredator responses. For example, cat presence induced hiding and freezing by rats in burrows, whereas cat odour alone made the rats leaving the burrows (Blanchard and Blanchard 1989). Rabbits showed a physiological stress response only in physical predator presence but not in sole presence of predator odours (Monclus et al. 2006, 2009). Physical presence of the predator and with that the possibility to interact with prey complicates interpretation of threat-sensitive behavioural changes for mainly two reasons. First, if predators are able to encounter and kill prey, supposed behavioural shifts observed in surviving prey may be due to selective predation of those individuals, which are innately less well capable of recognizing the predator and/or are less well able to respond to predation risk. In our study, the fate of IG prey (dead, survived) had no influence on the behavioural traits measured in the first time period of the experiment (where all were alive), excluding an important effect of selective predation on prey behaviour. Second, prey may respond differently to the physical presence of and interaction with two predator species, independent of the associated predation risk. For example, non-predatory animals may behave similarly (for example in motion patterns) as high-risk predators but differently from low-risk predators. In such a case, differing prey responses could not unambiguously be interpreted as threat-sensitive responses. However, differing responses of prey to two predator species, for example increase in running speed, may be interpreted as interspecific threat-sensitive antipredator responses, if the predator species differ in the risk posed to prey but do not differ in the corresponding behavioural trait, i.e. have the same running speed, or differ in opposite directions, i.e. the low-risk predator runs faster than the high-risk predator. Such scenarios occurred for several traits measured in our study. For interpretation of threat-sensitivity we thus analysed every behavioural trait of prey in light of the corresponding behavioural trait of the predator.

### Species-specific IG prey responses

Most behavioural traits of the IG prey larva of *A. andersoni* were unaffected by predator presence. Angular velocity and time spent moving away from the IG predator, however, were higher in presence of the high-risk IG predator *N. californicus.* The corresponding behavioural traits did not differ between the low and high-risk IG predator, but the fact that IG prey spent more time in spatial proximity of the high- than the low-risk IG predator indicates that these behavioural changes were not necessarily associated with the level of predator threat. Spatial proximity is defined as the distance where the IG predator can grasp the IG prey. The most likely explanation is that *A. andersoni* larvae did not distinguish between the two predators in avoiding their proximity but the stronger IG predator *N. californicus* stayed closer to them than *P. persimilis*. The high angular velocity of IG prey in the presence of the high-risk IG predator may indicate that IG prey is rapidly turning away from the encountering predator. Schausberger (2003) reported that the larva of the predatory mite *Galendromus occidentalis* (Acari: Phytoseiidae) displayed a typical side-kick with the caudal end of their body after an encounter with a conspecific aggressor. It could be that *A. andersoni* larvae are able to respond in a similar way or evolved other defence behaviours but this remains to be tested.

Intraguild predator presence induced several behavioural shifts in the IG prey larvae of *N. californicus* including higher velocity, longer distance moved and higher activity combined with a reduction in directional changes. *N. californicus* larvae responded to the high-risk IG predator with positive orthokinesis (increase in velocity) and negative klinokinesis (decrease in turning rates) simultaneously, which is characteristic for escape behaviours by straight forward movements to increase the distance between predator and prey (Furuichi 2002; Armsworth et al. 2005). In the presence of the low-risk IG predator *P. persimilis,* *N. californicus* larvae covered a longer distance at low velocity, which may indicate a response after disturbance or detection by the predator: encounters with the low-risk IG predator resulted in changes of the resting site. None of these behavioural IG prey shifts unambiguously indicates threat-sensitivity because the corresponding traits also differed between the low- and high-risk IG predator (velocity, distance moved, activity) or the behavioural shift of IG prey did not differ between the two predator species (turning angle, meander). However, *N. californicus* larvae were less frequent in spatial proximity of the high- than the low-risk IG predator. Theoretically, such a result may be caused by an avoidance response of the IG predator, IG prey or both. However, the high-risk IG predator *A. andersoni* is an aggressive IG predator attacking IG prey larvae immediately after detection (Schausberger and Croft 2000b; Walzer and Schausberger 2011a). We also observed that *A. andersoni* tried to chase *N. californicus* larvae after almost every encounter, which was rarely the case in the low-risk predator *P. persimilis*, making avoidance behaviour of the IG predator unlikely. Obviously, *N. californicus* larvae avoided more strongly to stay close to the high- than the low-risk IG predator and intensified this behaviour in the course of time. The time spent moving away from the IG predators did not differ but higher velocity and activity in presence of the high-risk IG predator allowed *N. californicus* larvae to more effectively reduce the likelihood of encounters with the high- than the low-risk IG predator. These findings indicate that *N. californicus* larvae were able to distinguish between the low- and high-risk IG predator, resulting in more pronounced spatiotemporal avoidance of the high-risk IG predator.

Every time, distance and path parameter of the IG prey larva of *P. persimilis* was affected by the presence of the IG predators. Differential responses to the predators in activity and avoidance behaviour of *P. persimilis* larvae suggest threat-sensitivity because the corresponding behavioural traits did not differ between the low- and high-risk IG predators *N. californicus* and *A. andersoni*. *Phytoseiulus persimilis* larvae showed two distinct strategies in relation to predation risk. In the presence of the low-risk predator, they covered a larger distance at higher velocity and increased their turning rates, which can be interpreted as escape behaviour by frequent directional changes. Conversely, the high-risk IG predator triggered a decrease in distance moved, velocity and activity, but an increase in turning angles and meander in IG prey. Furthermore, *P. persimilis* larvae avoided the leaf margin more strongly in presence of the high-risk IG predator and spent more time in moving away from them as compared to the presence of the low-risk predator.

### Prey vulnerability and anti-IG predator response intensity and complexity

The degree of complexity in and intensity of anti-IG predator responses (primary, i.e. avoid being detected, versus secondary, i.e. avoid being captured after detection, behavioural shifts) of the three IG prey species corresponds to their vulnerability in IGP, which in turn corresponds to the maternal investment in oviposition decisions (Walzer and Schausberger 2011a). IGP risk of larvae is an important factor influencing the oviposition site selection in the phytoseiid species looked at here (Walzer et al. 2006, Walzer and Schausberger 2011a). The six-legged larvae are less mobile than the later eight-legged nymphal stages and, because they do not have to feed to proceed with development (Schausberger and Croft 1999), commonly stay in the natal sites chosen by their mothers. The larva of *A. andersoni* is the least vulnerable IG prey species to fall victim to a predator within this guild. Consequently, *A. andersoni* females did not avoid placing their eggs in IG predator environments (Walzer and Schausberger 2011a). Moreover *A. andersoni* larvae more or less ignored the low-risk IG predator and responded to the high-risk IG predator only after being detected. *A. andersoni* apparently more relies on secondary antipredator mechanisms rather than avoid being detected by the predator. In contrast to *A. andersoni* females, *N. californicus* and *P. persimilis* avoided placing their eggs in prey patches containing cues of IG predators (Walzer and Schausberger 2011a). However, selective egg placement is not the only anti-IG predator strategy to expect, because IG predators may invade or return to the prey patch after oviposition by IG prey. In such a scenario the larva, which is not guarded or defended by its mother, should be able to adopt a primary or secondary anti-IGP mechanism. Our study suggests that both *N. californicus* and *P. persimilis* larvae mainly avoided being detected by the IG predators, but the type and intensity of their responses correlated with their differential vulnerability to IGP. Analogous to their mothers (Walzer and Schausberger 2011a), the moderately vulnerable *N. californicus* larva was little affected by the low-risk IG predator but tried to escape the high-risk IG predator, which in turn reduced the time spent in vicinity of the high-risk IG predator, decreasing the likelihood of an attack. The mothers of the highly vulnerable *P. persimilis* larvae responded to both low- and high-risk IG predators by preferential yet threat-sensitive egg deposition in predator free prey patches (Walzer and Schausberger 2011a). Similarly, *P. persimilis* larvae responded differently to the low- and high-risk IG predators, which ultimately reflects a threat-sensitive adaptation and proximately is determined by the motion paths of the IG predators. The activity levels of the low- and high-risk IG predators *N. californicus* and *A. andersoni* were similar but the latter covered much longer distances at higher velocity. An escape response as indicated by the higher activity levels of *P. persimilis* larvae in the presence of the low-risk IG predator, seems adequate to reduce the chances of being encountered and detected by a more slowly running predator. However, such a strategy would be counterproductive under the threat of a quickly running high-risk IG predator. Thus, *P. persimilis* larvae decreased their activity to avoid temporally and spatially encounters with the high-risk IG predator.

In summary, we assume that interspecific threat-sensitive IG prey responses should have involved in predator guilds where the guild members coexist for extended time periods. Theoretical support for this assumption comes from an IGP model recently developed by Urbani and Ramos-Jiliberto (2010), who integrated adaptive prey behaviour as threat-sensitive component in the model calculations, increasing the probability of persistence of both IG predator and IG prey as compared to most other IGP models.
